# Preparation and Characterization of Pazopanib Hydrochloride-Loaded Four-Component Self-Nanoemulsifying Drug Delivery Systems Preconcentrate for Enhanced Solubility and Dissolution

**DOI:** 10.3390/pharmaceutics14091875

**Published:** 2022-09-05

**Authors:** Seung Ah Choi, Eun Ji Park, Jun Hak Lee, Kyoung Ah Min, Sung Tae Kim, Dong-Jin Jang, Han-Joo Maeng, Sung Giu Jin, Kwan Hyung Cho

**Affiliations:** 1 College of Pharmacy and Inje Institute of Pharmaceutical Sciences and Research, Inje University, Gimhae 50834, Korea; 2 Department of Nanoscience and Engineering, Inje University, Gimhae 50834, Korea; 3 Department of Bio-Health Technology, College of Biomedical Science, Kangwon National University, Chuncheon 24341, Korea; 4 College of Pharmacy, Gachon University, Incheon 21936, Korea; 5 Department of Pharmaceutical Engineering, Dankook University, 119 Dandae-ro, Dongnam-gu, Cheonan 31116, Korea

**Keywords:** pazopanib hydrochloride, pH dependence, four-component self-nanoemulsifying drug delivery system SNEDDS, dissolution

## Abstract

The aim of this study was to develop a four-component self-nanoemulsifying drug delivery system (FCS) to enhance the solubility and dissolution of pazopanib hydrochloride (PZH). In the solubility test, PZH showed a highly pH-dependent solubility (pH 1.2 > water >> pH 4.0 and pH 6.8) and was solubilized at 70 °C in the order Kollisolv PG (5.38%, *w*/*w*) > Kolliphor RH40 (0.49%) > Capmul MCM C10 (0.21%) and Capmul MCM C8 (0.19%), selected as the solubilizer, the surfactant, and the oils, respectively. In the characterization of the three-component SNEDDS (TCS) containing Kolliphor RH40/Capmul MCM C10, the particle size of dispersion was very small (<50 nm) and the PZH loading was 0.5% at the weight ratio of 9/1. In the characterization of FCS containing additional Kollisolv PG to TCS, PZH loading was increased to 5.30% without any PZH precipitation, which was 10-fold higher compared to the TCS. The optimized FCS prepared with the selected formulation (Kolliphor RH40/Capmul MCM C10/Kollisolv PG) showed a consistently complete and high dissolution rate (>95% at 120 min) at four different pHs with 1% polysorbate 80, whereas the raw PZH and Kollisolv PG solution showed a pH-dependent poor dissolution rate (about 40% at 120 min), specifically at pH 6.8 with 1% polysorbate 80. In conclusion, PZH-loaded FCS in this work demonstrated enhanced solubility and a consistent dissolution rate regardless of medium pH.

## 1. Introduction

Pazopanib hydrochloride (PZH) is a tyrosine kinase inhibitor (TKI) indicated for the targeted treatment of advanced renal cell carcinoma (RCC) and advanced soft tissue sarcoma [1]. RCC is one of the most common malignant tumors occurring in the kidney and ranks within the top 10 for male and female cancer diagnoses [2]. PZH inhibits vascular endothelial growth factor receptor and platelet-derived growth factor receptor in cancer cells, thereby inhibiting the formation of new blood vessels [1].

**Figure 1:**
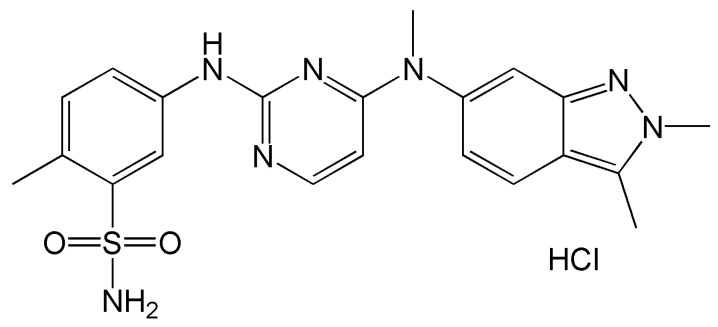
Structure of pazopanib hydrochloride.

PZH is classified as a BCS Class II crystalline solid powder that exhibits poor water solubility, high permeability, and low oral bioavailability of 14–39% [3]. The maximum daily dose (MDD) for the commercial product (Votrient, Novartis, Basel, Switzerland) is large enough to reach 800 mg [4]. This high dose has a correlation with hepatotoxicity and is a burden for patients; hence, the MDD should be mitigated with any alternative formulations [5]. PZH is expected to show a highly pH-dependent solubility since it has basic structural groups such as pyrimidine and sulfonamide, as shown in Figure 1 [6]. This pH-dependent solubility of PZH is expected to cause dissolution variability according to the medium pH [7,8]. Thus, there is a need to develop PZH formulations that enable enhanced solubility and dissolution rate regardless of medium pH.

A pharmaceutical food effect is described as a significant change in the rate or extent of drug absorption when a drug is administered in the fed state, compared to the fasted state [9]. The oral absorption of PZH is influenced and enhanced by food intake (twofold higher AUC in fed than in fasted states), and large variations in the blood concentration occur in the fed compared to the fasted state [10,11,12]. The exogenous lipid stimulates multiple secretory processes for lipid digestion in the gastrointestinal tract. Subsequently, lipid digestion products combine with endogenous lipids (i.e., bile salts, phosphatidylcholine, and cholesterol) to generate a range of colloidal solubilizing species which act as a reservoir for hydrophobic drug molecules [9,13]. This positive food effect of PZH can promote hepatotoxicity; therefore, to minimize toxicity through the food effect and variability in oral absorption, PZH is commercially indicated for consumption in a fasted state (at least 1 h before or 2 h after meals) [12]. In summary, there is still a need to develop improved formulations that reduce the effect of food intake.

Many studies have been conducted to increase the solubility and bioavailability of poorly soluble drugs. Representatively, nanoparticles, solid dispersions, self-nanoemulsifying drug delivery systems (SNEDDS), inclusion complexes, eutectic mixtures, and pulverization have been studied [14,15,16,17,18]. New attempts are also on the rise, such as the use of redox-sensitive self-immolating prodrug and electrospray methods to increase solubility and bioavailability [14,19]. A SNEDDS is a homogeneous, transparent solution composed of oil, surfactant, and solubilizer, which can be used as a carrier for poorly soluble drugs [20,21]. When administered orally, the SNEDDS spontaneously disperses in mild gastrointestinal motility to form O/W nanoemulsions. The formed O/W nanoemulsion can minimize external influences such as food intake or dissolution medium pH [22,23]. SNEDDS have demonstrated great potential in overcoming the food effect of various poorly water-soluble drugs such as cinacalcet, rivaroxaban, lurasidone, and cinnarizine [24,25,26]. SNEDDS can also reduce the effect of pH on dissolution since the drug molecule is encapsulated in the nanoemulsion particles and is less affected by the intestinal conditions, such as digestive secretion and medium pH [27]. In addition, they provide considerable advantages over liquid-state systems with regard to physicochemical stability, accurate dosing, ease of handling and storage, and improved patient compliance [9]. Thus, this is the most widely explored technology to enhance dissolution rate and bioavailability, as well as reduce the effect of food intake and dissolution medium pH, for poorly water-soluble drugs.

In this work, a new PZH-loaded four-component SNEDDS was developed for enhanced stability, solubility, and dissolution rate. The PZH solubility in various excipients and medium pH was determined, and optimized formulations were investigated in terms of PZH solubilization, SNEDDS dispersibility and particle size, and PZH precipitation suppression in SNEDDS. Moreover, a dissolution test in various pHs with or without solubilizer was performed to evaluate the enhanced dissolution rate. Transmission electron microscopy (TEM) images were obtained for the characterization of dispersed particles.

## 2. Materials and Methods

### 2.1. Materials

PZH was purchased from Hangzhou Royall Import and Export Co., Ltd. (Hangzhou, China). Kolliphor EL (polyoxyl 35 castor oil), Kolliphor HS15 (polyoxyl 15 hydroxystearate), Kolliphor RH40 (polyoxyl 40 hydrogenated castor oil), Kolliphor PS80 (polysorbate 80), Kollisolv PG (propylene glycol), Kollisolv MCT70 (medium-chain triglyceride), and Kollisolv PEG400 (polyethylene glycol 400) were provided by BASF (Ludwigshafen, Germany). Capmul MCM C8 (glyceryl monocaprylate) and Capmul MCM C10 (glyceryl monocaprate) were provided by ABITEC (Columbus, OH, USA). Castor oil was provided by DongYang Oil Chemical (Anseong, Korea). All other chemicals were of reagent grade and were used without further purification.

### 2.2. HPLC Conditions

The HPLC analysis of PZH in samples was conducted using a Waters 2695 HPLC system (Waters, Milford, MA, USA) equipped with a UV–Vis detector (Waters 2487, Waters, Milford, MA, USA). PZH was separated using a reversed-phase column (C18 column, 5 μm, 4.6 × 150 mm) (Osaka Soda, Osaka, Japan). The mobile phase was composed of 0.02 M ammonium acetate aqueous solution (pH 7), acetonitrile, and methanol (47/37/16, *v*/*v*/*v*). The HPLC analysis was performed with a flow rate of 1.0 mL/min. The injection volume was 10 μL, and UV detection was monitored at 260 nm. Data acquisition and processing were carried out using the Waters LC Solution software (2.0 version).

### 2.3. pH Solubility Test

The pH solubility of PZH was tested in pH 1.2 HCl/NaCl buffer, pH 4.0 acetate buffer, and pH 6.8 phosphate buffer and water. First, 10 mg of PZH was added to 10 mL of the prepared buffer and water in a vial, and stirring was performed at 500 rpm for 24 h. The mixture was aliquoted by 1 mL, put in a tube, and centrifuged at 15,000 rpm for 10 min (LZ-1730R, LABOGENE, Seoul, Korea). Then, 0.5 mL of supernatant was diluted 10-fold with a diluent (water and methanol; 50/50, *v*/*v*). The amount of PZH was analyzed using the HPLC condition as described in Section 2.2.

### 2.4. Solubility Test in Excipients

The solubility of PZH in various excipients such as surfactants, oils, and solubilizers was measured. The experiment was conducted at 50 or 70 °C to reach high solubility. Excess PZH powder (approximately 100 mg) was added to a vial containing 1 g of each excipient. Thereafter, the suspension in the vial was stirred in the oil bath at 50 or 70 °C for 24 h to achieve saturation. This sample was centrifuged at 15,000 rpm for 30 min (LZ-1730R, LABOGENE, Seoul, Korea), and the supernatant was diluted with a diluent (water and methanol; 50/50, *v*/*v*). The amount of PZH was analyzed using the HPLC condition as described in Section 2.2.

### 2.5. Preparation and Characterization of Two-Component Vehicle (TwCV)

In order to select specific oils and surfactants that exhibit the acceptable dispersed particle size, various TwCVs without PZH were prepared and evaluated. Oils and surfactants in a vial were combined at various weight ratios (1/9–9/1), heated to 70 °C in an oil bath, and mixed by stirring for 1 h. The samples were taken out and equilibrated at room temperature. The particle size of the dispersion was measured using the method as described in Section 2.8.

### 2.6. Preparation and Characterization of Three-Component SNEDDS (TCS)

For the preparation of the TCS, excess PZH powder (approximately 100 mg) was added to 5 g of the selected TwCV in a vial, heated to 70 °C in an oil bath, and mixed by stirring for 12 h. The mixture was centrifuged at 15,000 rpm for 30 min (LZ-1730R, LABOGENE, Seoul, Korea), and the supernatant was obtained as a TCS. TCS was diluted with a diluent (water and methanol; 50/50, *v*/*v*) and the amount of PZH was analyzed using HPLC condition as described in Section 2.2. The particle size of the dispersion was measured using the method described in Section 2.8.

### 2.7. Preparation and Characterization of Four-Component SNEDDS (FCS)

For the preparation of the FCS, excess PZH powder (approximately 500 mg) was added to 5 g of the three-component vehicle (ThCV; surfactant/oil/solubilizer) in a vial. This was followed by stirring at 70 °C in an oil bath for 12 h. The mixture was centrifuged at 15,000 rpm for 30 min (LZ-1730R, LABOGENE, Seoul, Korea), and the supernatant was obtained as an FCS. FCS was diluted with a diluent (water and methanol; 50/50, *v*/*v*) and the amount of PZH was analyzed using the HPLC condition as described in Section 2.2. The particle size of the dispersion was measured using the method described in Section 2.8. The visual observance of any PZH precipitation in the FCS was conducted on a daily basis.

### 2.8. Hydrodynamic Diameter Measurement

First, 100 mg of the sample was added to a vial with 2.9 mL of water. This was followed by vortexing for 1 min to completely disperse the samples. The particle size was determined using a particle size analyzer (NanoBrook 90Plus, Brookhaven instruments Corporation, Holtsville, NY, USA) at a wavelength of 659 nm and a scattering angle of 90°. The temperature was set to 25 °C, and the number of measurements was set at five cycles. The hydrodynamic diameter (hereinafter referred to as particle size) was measured in triplicate.

### 2.9. Dissolution Test

The dissolution test of the FCS, propylene glycol solution of PZH (PG solution), and raw PZH was performed using a 708-DS dissolution tester (Agilent Technologies Inc., Santa Clara, CA, USA). Each FCS equivalent amount of 50 mg PZH was filled into a gelatin capsule shell and then placed into a dissolution tester with a sinker. The dissolution test was performed under conditions of 50 rpm paddle speed in 900 mL of pH 1.2 medium, pH 4.0 medium, pH 6.8 medium, or water with or without 1% polysorbate 80 at 37 ± 0.5 °C. The samples were aliquoted in 5 mL increments with a syringe and collected at predetermined time intervals (5, 10, 15, 30, 45, 60, 90, and 120 min). The aliquoted sample was diluted 2-fold with a diluent (water and methanol; 50/50, *v*/*v*) and filtered through a 0.45 μm syringe filter. PHZ amount in the filtered sample was analyzed using the HPLC condition as described in Section 2.2.

### 2.10. Transmission Electron Microscopy (TEM) Analysis

The representative morphology and particle size of raw PZH and dispersed particles of FCS in pH 6.8 buffer with 1% polysorbate 80 were analyzed using a transmission electron microscope (Talos L120C, Thermo Fisher Scientific Inc., Waltham, MA, USA). Raw PZH in water and 50-fold dilution of FCS in pH 6.8 buffer with 1% polysorbate 80 was dispensed dropwise onto a carbon-coated TEM grid (FCF200-CU-50, Electron Microscopy Sciences, Inc., Hatfield, PA, USA) and dried for 30 min in a desiccator. Then, the prepared sample was scanned at the accelerating voltage of 120 kV.

## 3. Results and Discussion

### 3.1. pH Solubility

The solubility of PZH in various pHs was determined at room temperature (Table 1). The solubility at pH 1.2 was the highest with 682.64 ± 7.58 μg/mL, about 200-fold higher than that at pH 4.0 (3.00 ± 0.25 μg/mL) and pH 6.8 (2.64 ± 1.02 μg/mL). The solubility in water was 144.08 ± 2.56 μg/mL. The higher solubility in acidic pH 1.2 resulted from the full protonation and ionization of the basic structural groups such as indazole and pyrimidine, as their ionization constants (pKa = 2.1 and 6.4) were higher than the pH [6,28]. However, PZH is insoluble at pH above 6.8 because it is essentially in a completely non-ionized form due to its pKa value of 6.4 [6]. As the pH increases, the solubility decreases due to the decreased proportion of ionized forms. In addition, the PZH hydrochloride (PZH free base: HCl = 1:1) was solubilized without the effect of the buffer [29]. Thus, PZH exhibited a pH-dependent solubility (pH 1.2 >> water > pH 4.0 and pH 6.8) and was considered practically insoluble at all pHs.

### 3.2. Solubility in Excipients

**Figure 2:**
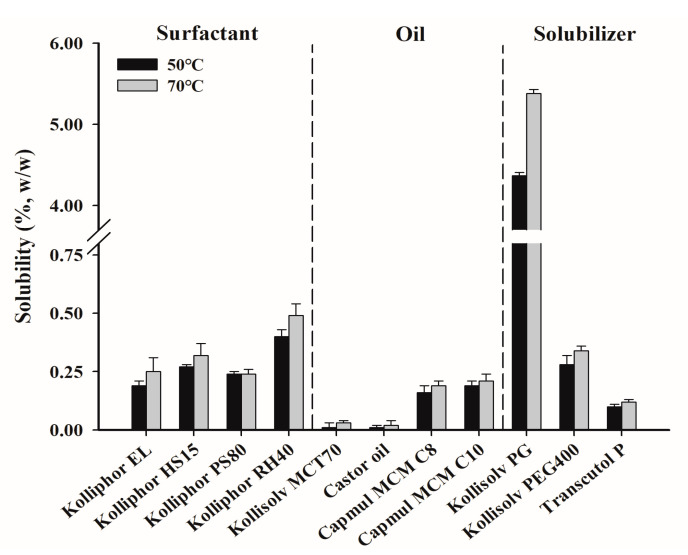
The solubility of PZH in various surfactants, oils, and solubilizers.

A PZH solubility test was performed at 50 °C and 70 °C to select a suitable surfactant, oil, and solubilizer for the composition of SNEDDS in Figure 2. The solubility at 70 °C was overall higher than that at 50 °C [30]. Among the solubilizers, the solubility of PZH in Kollisolv PG was the highest at 70 °C with 5.38 ± 0.05% (*w*/*w*), which was about 16-fold higher than that in Kollisolv PEG400 (0.34 ± 0.02%). Kollisolv PG was effective as a solubilizer since it showed the highest solubility. Among the surfactants, Kolliphor RH40 (0.49 ± 0.05%) showed relatively higher solubility than Kolliphor HS15 (0.32 ± 0.05%) and Kolliphor EL (0.25 ± 0.06%). Among the oils, Kollisolv MCT70 and castor oil showed a negligibly low solubility of <0.1%. Capmul MCM C8 and Capmul MCM C10 showed relatively higher solubility of 0.19 ± 0.02% and 0.21 ± 0.03%, respectively, with no significant difference between them. Therefore, Kollisolv PG as a solubilizer, Kolliphor RH40 as a surfactant, and Capmul MCM C8 and Capmul MCM C10 as oils were selected for further studies on the basis of solubility.

### 3.3. Characterization of Two-Component Vehicle (TwCV)

**Figure 3:**
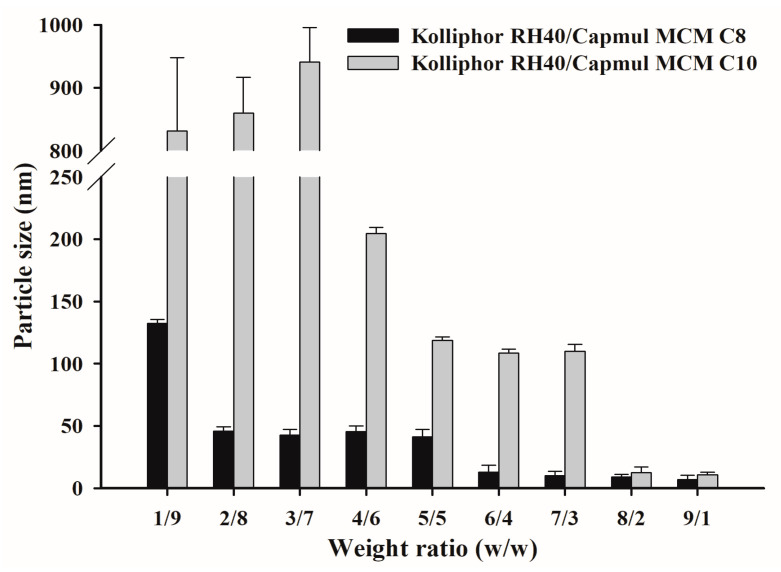
The particle size of TwCVs dispersed in water.

TwCV without PZH was characterized to select the appropriate oil between Capmul MCM C8 and Capmul MCM C10, as well as the suitable weight ratio for use (Figure 3). Capmul MCM C8 and Capmul MCM C10 were mixed with Kolliphor RH40 at a weight ratio of 1/9–9/1, and the particle size of dispersion was measured. In most TwCVs, as the weight ratio of surfactant increases, the particle size tends to decrease [31]. All TwCVs containing Capmul MCM C8 showed particle sizes of <150 nm. TwCVs containing Capmul MCM C10 showed a relatively larger particle size than those containing Capmul MCM C8 at the weight ratios of 1/9–7/3, but a similarly small particle size of <50 nm at the weight ratios of 8/2–9/1. The longer length of the carbon chain (C10 vs. C8) in Capmul MCM C10 would produce a more viscous vehicle, resulting in a larger particle size dispersion at most weights as compared to Capmul MCM C8. However, at the weight ratios of 6/4–9/1, both Capmul MCM C8 and Capmul MCM C10 showed a suitable particle size of <150 nm and almost transparent dispersion (not shown). Therefore, the TwCVs containing Kolliphor RH40 (surfactant) and Capmul MCM C8 and Capmul MCM C10 (oil) were selected at higher surfactant ratios of 6/4–9/1.

### 3.4. Characterization of Three-Component SNEDDS (TCS)

**Figure 4:**
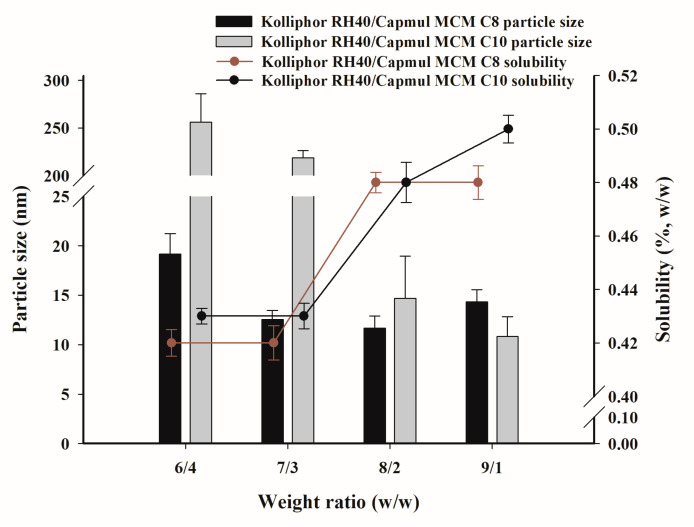
The saturated solubility of PZH in TCV and the particle size dispersed in water.

The saturation solubility of PZH and the particle size of dispersion were measured at the selected TwCV composition, as summarized in Figure 4. It was found that the saturated solubility of PZH was higher at weight ratios of 8/2 and 9/1 due to the higher solubility of surfactant (Kolliphor RH40) compared to oils (Capmul MCM C8 and C10), as shown in Figure 2. The solubility of PZH at 9/1 was 0.50 ± 0.01% for Kolliphor RH40/Capmul MCM C10 and 0.48 ± 0.01% for Kolliphor RH40/Capmul MCM C8. Kolliphor RH40/Capmul MCM C10 at 6/4 and 7/3 showed a slight increase in particle size (>200 nm) compared to the TwCV (<150 nm) due to the PZH loading [6]. However, a small particle size of <50 nm was observed at 8/2 and 9/1. For Kolliphor RH40/Capmul MCM C8, the particle size of dispersion was small (<50 nm) at weight ratios of 6/4–9/1, and there was no effect of PZH loading on the particle size. As a result, the TCS composed of Kolliphor RH40/Capmul MCM C8 and Kolliphor RH40/Capmul MCM C10 showed a small particle size and relatively high PZH loading at a weight ratio of 9/1; thus, it was subsequently used for the preparation of the FCS.

### 3.5. Characterization of Four-Component SNEDDS (FCS)

The FCS was prepared and evaluated with various ThCVs (three-component vehicles) composed of TwCVs and Kollisolv PG at weight ratios of 1/9–9/1, as shown in Table 2. The saturated solubility increased proportionally with the increase in weight ratio of Kollisolv PG that provided the highest solubilization of PZH. The maximum solubility in FCS10 (5.69%) was about 11-fold higher compared to the TCS (≤0.50%). The FCS revealed an optimal particle size (<50 nm), with the exception of FCS1. Thus, the FCS was also optimized in terms of PZH loading and particle size of dispersion. However, the saturated solubility of PZH obtained at 70 °C indicated the potential of PZH precipitation during storage at room temperature; hence, the precipitation occurrence was investigated over 50 days. In the comparison of FCS1–9 and FCS10–18, Capmul MCM C10 inhibited PZH precipitation effectively compared to Capmul MCM C8. The saturated and solubilized PZH in FCS was stabilized by ThCV containing Capmul MCM C10. For reference, PZH saturated only in Kollisolv PG resulted in precipitation within 3 days and was physically unstable (data not shown). The upper solubility limit with no precipitation was 2.10 ± 0.06% for FCS5 and 5.30 ± 0.13% for FCS11. As a result, FCS11–18 were ultimately optimized in terms of PZH loading, dispersibility and particle size, and physical stability.

### 3.6. Dissolution Test

**Figure 5:**
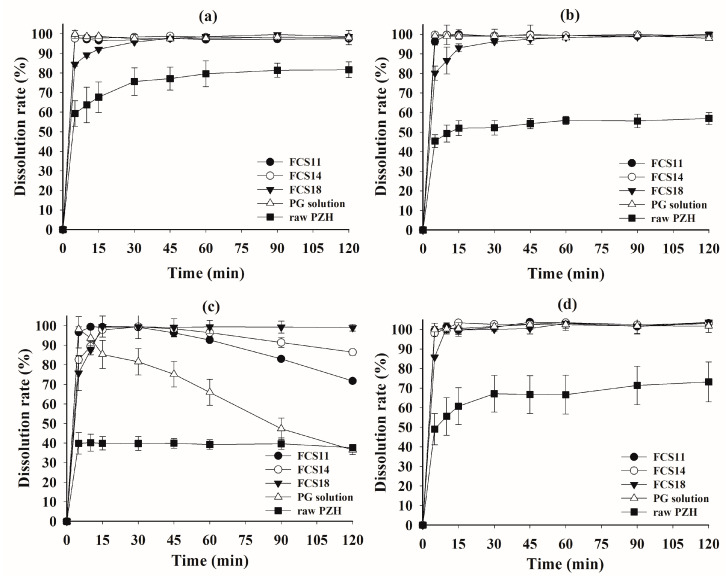
Dissolution profiles of FCS formulations and raw PZH in pH 1.2 buffer (**a**), pH 4.0 buffer (**b**), pH 6.8 buffer (**c**), and water (**d**) with 1% polysorbate 80.

**Figure 6:**
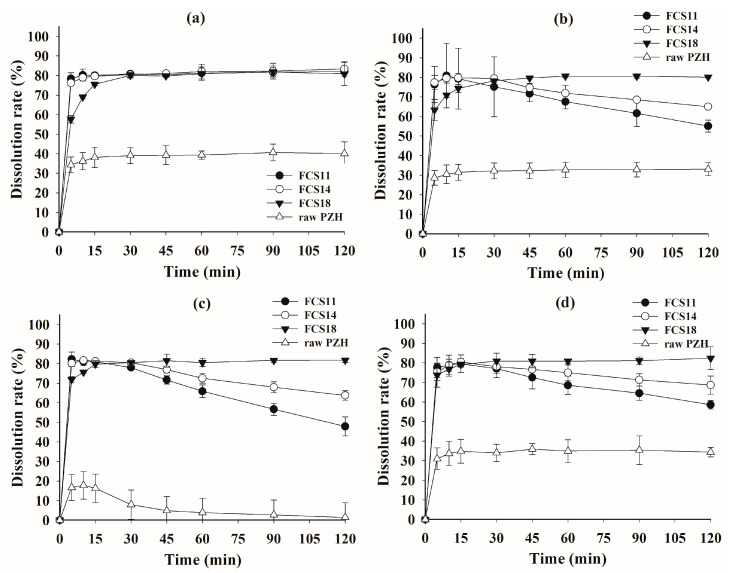
Dissolution profiles of FCS formulations and raw PZH in pH 1.2 buffer (**a**), pH 4.0 buffer (**b**), pH 6.8 buffer (**c**), and water (**d**).

A dissolution test was performed on raw PZH, as well as FCS11, FCS14, FCS18, and the Kollisolv PG solution of PZH (PG solution), in pH 1.2 buffer, pH 4.0 buffer, pH 6.8 buffer, and water, with or without 1% polysorbate 80 as solubilizer (Figure 5 and Figure 6). In pH 1.2 buffer, pH 4.0 buffer, and water with 1% polysorbate 80, all FCSs showed a complete dissolution rate of >95% within 30 min, whereas raw PZH showed a low steady-state dissolution rate (81.68% at pH 1.2, 56.96% at pH 4.0, and 73.16% in water) within 120 min. There was significant discrimination between FCSs and the other samples (PG solution and raw PZH) in pH 6.8 buffer, whereby PZH solubility was very hard to maintain. Raw PZH and PG solution showed very poor dissolution rates of 39.27 and 65.95% at 60 min, respectively. Moreover, PG solution decreased sharply to 36.53% from the initial complete dissolution rate, indicating that it underwent immediate recrystallization and precipitation upon exposure to the external high-pH medium. However, FCS11, FCS14, and FCS18 showed high dissolution rates > 90% until 60 min, which were slightly decreased or maintained at 71.73, 86.43, and 98.97% at 120 min, respectively. The FCSs formed a nanoemulsion immediately after dispersion in medium, which reduced the influence of external pH and pressure on precipitation [30,32]. Among FCSs, a decrease in dissolution rate from 30 min to 120 min was observed in the order FCS11 (5.30% PZH loading) > FCS14 (2.19% PZH loading), whereas there was no decrease observed for FCS18 (0.53% PZH loading). A high PZH loading in FSCs favored PZH precipitation under pH stress, but FCS significantly improved the solubility and dissolution rate compared to raw PZH and PG solution [33].

In the dissolution test without polysorbate 80 (Figure 6), the raw PZH showed a very poor steady-state dissolution rate of <40% at all pH due to the absence of the solubilizer, i.e., polysorbate 80. Although the initial peak dissolution rate for FCS at 5 min was around 80%, it was much better than that of raw PZH. The peak dissolution rate in all FCSs was maintained at pH 1.2, but there was a decrease for FCS11 and FCS14 at pH 4.0 and pH 6.8, as well as in water. The higher PZH loading in both FCSs clearly resulted in a decrease in dissolution rate compared to FCS18. However, the FCS maintained the peak dissolution until 30 min, followed by a slight decrease at pH 4.0 and in water. These optimized FCSs reduced the effect of the medium pH on dissolution by spontaneously forming a stable nanoemulsion with high drug loading and physical stability, as well as minimum PZH precipitation [34]. The FCSs displayed a significantly better area under the dissolution curve at all pHs compared to raw PZH, indicating the enhanced solubility and dissolution rate in the wide range of pH.

In the previous study by Choi et al. (2020) and Herbrink et al. (2018), the dissolution patterns of the commercial product and the raw PZH were similar. In particular, the commercial product showed a final dissolution rate of less than 20% in pH 6.8 buffer [3,7]. Therefore, it was considered that FCSs exhibited excellent increased dissolution results compared to the commercial product. In addition, the increased dissolution rate can be expected to increase the bioavailability [7].

### 3.7. TEM Analysis

**Figure 7:**
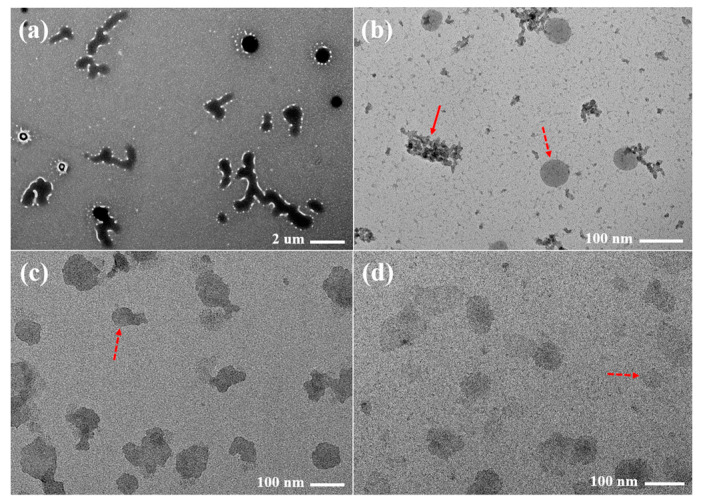
Transmission electron microscopy (TEM) images of raw PZH (**a**) dispersed in water, FCS11 (**b**), FCS14 (**c**), and FCS18 (**d**) dispersed in pH 6.8 buffer with 1% polysorbate 80.

The representative TEM images of the raw PZH dispersed in water and FCSs dispersed in pH 6.8 buffer with 1% polysorbate 80 are presented in Figure 7. The raw PZH had an irregular shape of <10 μm in size and showed an opaque internal state. In contrast, the representative single spherical and translucent nanoemulsion droplets (red dotted arrow) of <100 nm were observed in FCS11, FCS14, and FCS18, while aggregated particles (red arrow) that appeared to form PZH precipitates were also observed in FCS11. No precipitation was observed for FCS14 and FCS18. The major droplets for F14 and F18 were soft and crushed together compared to F11. The small particle size of the FCS18 with no PZH precipitation could explain the high dissolution rate [35].

## 4. Conclusions

In this study, a four-component self-nanoemulsifying drug delivery system (FCS) was developed to enhance the solubility and dissolution of pazopanib hydrochloride (PZH). A new FCS formulation was constructed through a systematic approach to evaluate the solubility of PZH, precipitation occurrence, particle size of dispersion, and dissolution rate in various pHs. The optimized FCS composed of Kolliphor RH40/Capmul MCM C10/Kollisolv PG resulted in high solubilized loading up to >5%, a dispersion particle size of <50 nm, good physical stability without precipitation, and a consistently high dissolution rate (>90% at 60 min) in various pHs with 1% polysorbate 80. In conclusion, the PZH-loaded FCS in this work can be suggested as a promising formulation candidate with enhanced solubility and stability, as well as a consistent dissolution rate, regardless of pH.

## Figures and Tables

**Table 1:** The pH solubility of PZH in various pH. Data are presented as the means ± standard deviation (*n* = 3).

Test Solution	Solubility (μg/mL)
pH 1.2 buffer	682.64 ± 7.58
pH 4.0 buffer	3.00 ± 0.25
pH 6.8 buffer	2.64 ± 1.02
water	144.08 ± 2.56

**Table 2:** The PZH solubility, precipitation occurrence, and particle size of water dispersion, for the FCS.

FCS	ThCV Composition (%, *w*/*w*)				PZH Solubility (%, *w*/*w*)	Precipitation Occurrence	Particle Size of Water Dispersion (nm)
	Kolliphor RH40	Capmul MCM C8	Capmul MCM C10	Kollisolv PG			
FCS1	9	1	0	90	4.94 ± 0.05	P	84.07 ± 30.80
FCS2	18	2	0	80	4.44 ± 0.10	P	15.07 ± 2.81
FCS3	27	3	0	70	3.97 ± 0.06	P	9.70 ± 0.44
FCS4	36	4	0	60	3.02 ± 0.07	P	13.03 ± 3.28
FCS5	45	5	0	50	2.10 ± 0.06	N	11.33 ± 2.35
FCS6	54	6	0	40	1.75 ± 0.12	N	10.27 ± 1.10
FCS7	63	7	0	30	0.86 ± 0.10	N	13.60 ± 3.11
FCS8	72	8	0	20	0.66 ± 0.05	N	12.55 ± 0.07
FCS9	81	9	0	10	0.50 ± 0.08	N	13.60 ± 0.89
FCS10	9	0	1	90	5.69 ± 0.06	P	16.33 ± 1.83
FCS11	18	0	2	80	5.30 ± 0.13	N	14.47 ± 0.31
FCS12	27	0	3	70	3.34 ± 0.11	N	13.57 ± 1.59
FCS13	36	0	4	60	2.66 ± 0.08	N	15.28 ± 1.74
FCS14	45	0	5	50	2.19 ± 0.04	N	16.30 ± 2.90
FCS15	54	0	6	40	1.44 ± 0.09	N	16.73 ± 4.60
FCS16	63	0	7	30	0.75 ± 0.15	N	12.57 ± 2.10
FCS17	72	0	8	20	0.57 ± 0.06	N	10.70 ± 3.87
FCS18	81	0	9	10	0.53 ± 0.07	N	10.43 ± 3.99

Data are presented as the mean ± standard deviation (*n* = 3). P: precipitation within 5 days; N: no precipitation within 50 days.
